# Editorial: Exercise Friend or Foe? For the Management of Oxidative Stress in Health and Diseases

**DOI:** 10.3389/fphys.2022.881197

**Published:** 2022-03-23

**Authors:** Anand Thirupathi, Yaodong Gu, Huw David Wiltshire, Ricardo A. Pinho

**Affiliations:** ^1^Faculty of Sports Science, Ningbo University, Ningbo, China; ^2^Cardiff School of Sport and Health Sciences, Cardiff Metropolitan University, Cardiff, United Kingdom; ^3^Laboratory of Exercise Biochemistry in Health, Graduate Program in Health Sciences, School of Medicine, Pontifícia Universidade Católica do Paraná, Curitiba, Brazil

**Keywords:** oxidative stress, exercise, free radicals, muscles, oxidants and antioxidants

Since the initial findings of free radicals existence in the biological samples (Commoner et al., 1954) revealed the negative effects of free radicals within the hypothesis of a free radical theory of aging, the specific role of exercise-induced free radicals in the muscle functions gained much interest among the scientific community in various ways such as defining the level of free radicals that impacts physiopathological functions and subsequent use of antioxidants (Davies et al., 1982; Jackson et al., 1983). As the view of the negative effects of exercise-induced free radicals has been changed, which does not mean these free radicals are non-toxic. Instead, it shows the requirement to define the level of free radicals, their production site, and transience within the skeletal muscle and to other muscles. Apart from these major flaws in terms of free radical biology and exercise physiology, the benefit of exercise is obvious. As a result, the recommendation of physical exercise has been implemented for a healthy life. In recent years, physical exercise has been suggested for disease management programs as it promotes several socioeconomic and biological benefits, including psychological, cognitive, physiological, molecular, and biochemical benefits. However, these biological benefits are individualized according to the human genetic setup (Pinho et al., 2019), and it depends on how the body efficiently overcomes the redox changes during exercise. This is mainly achieved through designing proper exercise protocols considering the intensity, duration, frequency, and type of exercise. Although the antioxidant effects and detrimental effects of oxidants have been predominantly reported previously, the role of oxidants in diverse physiological processes during exercise has also been highlighted by scientists. Exercise could be the additional factor at the same time needed to keep oxidant levels within a bearable threshold for cells; otherwise, it causes redox imbalance and consequent cellular damage (Powers et al., 2020). The performance of long-term exercises with adequate intensities activates several adaptive signaling pathways whose role is to maintain an adaptive response of cells and organisms against oxidative stress induced by exercise, biological response conceptually defined as hormesis (Radak et al., 2008). The long-term effects of physical exercise are dependent on intracellular communication and synergy between organs and tissues (Pinho et al., 2019); however, each exercise program can lead to different cellular responses and induce local and systemic oxidative stress, and how this is accomplished in a coordinated manner required further investigation.

This current Research Topic provided eight articles dealing with the effects of different types of exercises on reducing oxidative stress in pathological conditions and maintaining ROS in physiological conditions. These manuscripts will expand knowledge in the area and help obtain better treatment for several chronic diseases due to redox imbalance being the core of many diseases. Within this context, a study provided evidence of aerobic exercise in treating cardiac dysfunction by improving antioxidants capacity and decreasing ROS during the advanced stage of uncontrolled arterial hypertension is a major cause of cardiac remodeling and consequent heart failure (Pagan et al.). These results modify various signaling related to myocardial dysfunctions such as JNK, p38, NF-kB, and ERK. Another article suggested that exercise prevents oxidative stress-induced consequences in the lungs after exposure to particulate matter. The exercise reduced the lung inflammatory response and oxidative stress (So et al.), but the benefits depend on exercise protocols and individuals' adaptive capacity. In the case of higher intensity, a long-term exercise induces an adaptive response against ROS-induced consequences. However, it is not suitable in all cases, such as aging and chronic disease conditions (excessive prooxidant environment) where the adaptive signaling is almost blunt or failed to activate. In such cases, antioxidants could be recommended along with performing exercise because even a single bout of exercise aggravates the excessive prooxidant environment in aging and chronic diseased conditions. In this aspect, another article provided evidence of using hydrogen gas as an antioxidant in comparison to Vitamin C, and inhalation of hydrogen gas could effectively intoxicate exercise-induced ROS by activating PGC-1alpha, TFAM, and NRF-2 gene expression, while vitamin C blunts the mitochondrial biogenesis signaling (Chaoqun et al.), suggesting the careful selection and use of antioxidants. In addition, this Research Topic provided two systematic reviews and a meta-analysis which showed that a higher intensity of aerobic exercise exacerbates oxidative stress (Lu et al.). In contrast, moderate exercise had a better effect on ameliorating oxidative stress in aging and general people (Ye et al.; Pan et al.). Next, an illustrated review discussed the exercise effect on oxidative stress and diaphragm dysfunction, which may depend on exercise intensity, duration, and type of exercise (Zhang et al.). Another review article illustrated how physical exercise influences psychological, genetic markers (Wang et al.).

In summary, this special issue addressed several new aspects involving physical exercise in the management of diseases linked to oxidative stress. The adaption of physical exercise as part of the lifestyle effectively prevents and controls the progression of chronic diseases and increases longevity with quality of life. Although physical exercise is a non-invasive and successful human behavior in preventing and treating chronic diseases, its effects on redox biology are still poorly investigated and, therefore, deserve attention from the scientific community. It is necessary to encourage new special editions related to this topic and expand access to scientific advances for all. Scientists should invest their efforts in understanding the redox mechanisms induced by physical exercise. Finally, we thank all authors, reviewers, and editors for their extraordinary contribution to this topic.

## Author Contributions

AT, YG, HW, and RP wrote the article. All authors listed have made a substantial, direct, and intellectual contribution to the work and approved it for publication.

## Conflict of Interest

The authors declare that the research was conducted in the absence of any commercial or financial relationships that could be construed as a potential conflict of interest.

## Publisher's Note

All claims expressed in this article are solely those of the authors and do not necessarily represent those of their affiliated organizations, or those of the publisher, the editors and the reviewers. Any product that may be evaluated in this article, or claim that may be made by its manufacturer, is not guaranteed or endorsed by the publisher.
